# The Effect of Perceived Value on Consumers’ Repurchase Intention of Commercial Ice Stadium: The Mediating Role of Community Interactions

**DOI:** 10.3390/ijerph19053043

**Published:** 2022-03-05

**Authors:** Wenyu Zang, Yuhao Qian, Hemin Song

**Affiliations:** Sports Business School, Beijing Sport University, Beijing 100084, China; zangwenyu123@163.com (W.Z.); yuhao@bsu.edu.cn (Y.Q.)

**Keywords:** perceived value, mediation effect, commercial ice rinks, community interactions, repurchase intention

## Abstract

The 2022 Beijing Winter Olympics has created unprecedented opportunities for China’s commercial ice rinks, where improving consumers’ repurchase intention is essential for their high-quality development. This paper explores the mediation effect of community interactions between perceived value and commercial ice rink consumers’ repurchase intention based on the theory of perceived value. Through a questionnaire survey, we collected 347 valid questionnaire from consumers of commercial ice rinks. Based on a structural equation model, the results show that consumers’ perceived risk value significantly impacts community interactions and consumers’ repurchase intention. Perceived functional, social, and emotional values positively affect community interactions but have an insignificant impact on the consumers’ repurchase intention. Community interactions play a full mediation role between perceived emotional and social values and consumers’ repurchase intention; they play partially mediating role between consumers’ perceived risk value and repurchase intention and do not have mediating role between perceived functional value and consumers’ repurchase intention. Therefore, we provide some practical suggestions: extending commercial ice rink product lines, creating unique intellectual property systems, improving consumer sports career planning, and enhancing risk management.

## 1. Introduction

China’s ice and snow sports industry is booming with the issue of the “Opinions on Accelerating the Development of the Sports Industry and Promoting Sports Consumption” (No. 46) promulgated by the State Council and hosting of the 2022 Beijing Winter Olympics [1]. The industry will grow to one trillion Yuan by 2025, according to China’s “Winter Sports Development Plan (2016–2025)” [2]. According to the survey report on “Spurring 300 million People to Participate in Ice and Snow Sports” from the National Bureau of Statistics, since Beijing won the bid for the Winter Olympics in 2015, the number of people participating in snow and ice sports has reached 346 million, the participation rate of residents has reached 24.56%, and there are 654 standard ice rinks and 803 indoor and outdoor ski resorts. Ice and snow venues are essential carriers of the ice and snow sports industry. Commercial ice rinks popularize such venues [3], as they break through the time and space limitations of ice and snow sports and are of great significance to implementing the strategy of “expanding ice and snow sports into the South from the North, extending it to the West and introducing it to the East” [4]. Previous studies have shown that as a facility with a high rate of return, the commercial ice rink has the advantages of attracting popularity [5], and whether the advantages can be used depends on consumers’ demands for service quality, and the operation and management method of the commercial ice rink [6]. Obviously, how to retain consumers, thereby generating repurchase behavior, has become the key for a commercial ice rink to maintain its competitive advantage [7]. Nevertheless, the pervasive prominent phenomenon of “top-end concentration,” the lack of professional talent, and the cumbersome reservation procedures reduce consumers’ willingness to participate [3], hindering the high-quality development of commercial ice rinks. Thus, creating incentives to generate repurchase by commercial ice rink consumers is of significant theoretical and practical value.

The majority of academic research on commercial ice rinks focuses on the development of stadiums [6], existing problems [3], prospects [4], etc. There is a lack of research on improving consumers’ desire to repurchase. Although some studies have shown the importance of consumers’ perceived value on repurchase intention [8], the mechanism by which perceived value affects consumer repurchase intention, especially the role of community interactions, is rarely considered [9]. The impact of community interactions on consumption as a form of social interaction created by information dissemination within a community [10] has become the focus of research in consumer behavior [9,10,11]. Still, academia rarely analyzes the impact of perceived value on consumers’ repurchase intention regarding community interactions for commercial ice rinks (in the consumption of commercial ice rinks, purchasers are often separated from users. This study only focuses on consumers with independent consumption capacity. The consumers mainly include parents and some teenagers with consumer awareness).

This paper offers a different perspective on popularizing ice and snow sports by taking consumers’ perceived value of commercial ice rinks as the starting point, establishing a structural equation model with community interactions as the mediator, and analyzing the impact mechanism of consumers’ perceived value on repurchase intention.

### 1.1. Theoretical Foundations and Research Hypothesis

#### 1.1.1. The Theory of Perceived Value

The theory of perceived value holds that customer perceived value is the overall evaluation of the utility of a product or service based on weighing the perceived benefits against the cost of obtaining the product or service [12]. Many scholars have studied the influence of perceived value on consumers’ repurchase intention. Sheth et al. [13] believe that decision-making is affected by five consumption values: functional, social, emotional, epistemic, and conditional. This model seems inadequate in explaining consumers’ complex consumption behavior for the ever-changing consumer market. Sweeney and Soutar [14] argued that epistemic and conditional values should be excluded from the perceived value structure. Lu et al. [15] further pointed out that risk is essential in perceived value and examined the relationship between consumers’ perceived quality, risks, profit, satisfaction, etc., and their purchase intention. They also found that perceived risk has significantly negative correlations between repurchase intention and consumers’ satisfaction, which means perceived risk becomes a kind of threat to consumers. This paper builds on the existing literature and, while considering Chinese-based commercial ice rinks, categorizes consumers’ perceived value into four dimensions (functional, emotional, social, and risk perceptions), and discusses the relationship among consumer perceived value, community interaction, and repurchase intention.

#### 1.1.2. Perceived Value and Repurchase Intention

Perceived functional value represents consumers’ subjective perception of a commodity or service’s principal functions, utility, or physical attributes and is crucial for driving consumption [13,16]. The consumer’s functional value perception of sports venues plays a value-enhancing role in consumer experience [17], and service quality especially impacts consumers’ perceived value, attitude, and behavior [18]. Consumers’ functional value perception of commercial ice rinks involves factors such as hardware facilities, coach resources, and service variety [6]. Excellent functional value perception improves consumer satisfaction, which positively impacts customers’ repurchase intention.

The perceived social value represents conventional consumer perceptions based on demographic, social, economic, or ethnocultural associations [13]. Sports-venue-related research shows that consumers are sensitive to the social responsibility of commercial ice rink operators, and the positive effects such as public welfare and humanistic values the operators bring to surrounding communities impact consumers’ perceived social value [19]. Wu et al. [20] recently indicated that corporate social responsibility has a largely positive effect going through brand identity and brand trust, and then affecting the decision to repurchase. Consequently, consumers’ satisfaction with a company increases when they perceive that it has fulfilled its social responsibilities, which translates into repurchase intention [21].

Perceived emotional value comprises the specific emotional connections generated when consumers use a product or service, impacting consumer experience and behavior by triggering emotions [13]. When consuming sports, the value of consumers’ emotional perception is mainly reflected in their subjective acceptance of a sport. Commercial ice rink consumers’ sporting skill level and preference will lead to strong emotional value perception, prompting them to form sports attachment and enhancing their willingness to repeat consumption.

Perceived risk value comprises the potential risks that consumers perceive in a product or service [22]. Perceived risks negatively impact consumer decision-making [23]. Jeong and Jo [24] believe that risk perception positively affects hesitation, and hesitation negatively affects purchase behavior intention. Like any sport, ice sports involve risks such as athletes’ skating speeds posing potential safety risks [25]. Simultaneously, factors such as ice rink operators’ rates, training methods, and safety measures may provoke consumers to question expected results [25,26,27]. Hence, higher consumer risk perception partially hampers repurchase intention.

This paper proposes the following hypotheses:

**Hypothesis** **1.** *Perceived functional value has a significant positive impact on commercial ice rink consumers’ repurchase intention*.

**Hypothesis** **2.** *Perceived social value has a significant positive impact on commercial ice rink consumers’ repurchase intention*.

**Hypothesis** **3.** *Perceived emotional value has a significant positive impact on commercial ice rink consumers’ repurchase intention*.

**Hypothesis** **4.** *Perceived risk value has a significant negative impact on commercial ice rink consumers’ repurchase intention*.

#### 1.1.3. Perceived Value and Community Interactions

As an essential concept in consumer behavior research, community interaction and its applications have always attracted attention. Community interactions usually refer to the interactions among community members, between the organizer and members, and between the organizer and community [28]. Generally speaking, consumers’ purpose in community interactions is to obtain relevant information [29].

Perceived functional value can result directly in continuous participation [30]. Consumers will be better positioned to accurately evaluate the functions of commercial ice rinks when they have a comprehensive and in-depth perception of services and facilities, which is conducive to the extensive interactions among community members and improves interaction quality [31].

The improvement of perceived social value is closely related to community connections. It is conducive to maintaining robust relationships within a community [32]. Sports venues may increase their patrons’ sense of belonging and interactivity by introducing social value formats such as cultural and artistic performances, religious activities, and leisure and recreational activities [33]. Similarly, improving perceived social value positively impacts community interactions for commercial ice rinks.

Perceived emotional value involves achievement, social, and entertainment emotions [34]. Achievement emotions are consumers’ self-satisfaction perceived when acquiring new knowledge and skills, which can inspire habits of relying on the community to gain knowledge [35]. Social emotions help members establish and maintain their relationship networks through community building. The enjoyable and exciting attributes of the commercial ice rink communities are also reflected through the perception of entertainment emotions, prompting members to stay in the community and continue interacting [36]. Thus, emotional value perception positively affects ice rink consumers’ initiating or engaging in community interactions [37,38].

The perceived risk value of consumers concerning commercial ice rinks is usually related to infrastructure [39]. Ice sports carry injury risks. Increased risk perception can easily create an unpleasant emotional experience, leading to more intense expectations for anonymous interactions [40], which are often independent of the ice rink community interactions, resulting in reduced community interactivity [41].

This paper proposes the following:

**Hypothesis** **5.** *Perceived functional value has a significant positive impact on community interactions*.

**Hypothesis** **6.** *Perceived social value has a significant positive impact on community interactions*.

**Hypothesis** **7.** *Perceived emotional value has a significant positive impact on community interactions*.

**Hypothesis** **8.** *Perceived risk value has a significant negative impact on community interactions*.

#### 1.1.4. The Mediation Effect of Community Interactions

Studies have shown that consumer participation in community interactions positively affects brand preference formation [42], which significantly impacts purchase intention [43,44]. In the sports community, members will form group identities through community behaviors such as communication, collaboration, mutual assistance, and experience sharing, impacting consumer attitudes and behaviors [45].

When consumers consume in a commercial ice stadium for the first time, they will establish perceived value in relation to the commercial ice stadium. Many studies have shown that the perceived value is positively correlated with consumers’ sense of identity and loyalty to a commercial ice stadium, and the improvement of sense of identity and loyalty will promote a high level of community interactions [38,46,47,48]. The level of community interactions will further affect the repurchase intention of consumers [49]. In the final analysis, there is an indirect effect between perceived value and community interactions. The previous hypothesis has assumed that perceived value has a significant effect on repurchase intention. Thus, this paper proposes the following:

**Hypothesis** **9.** *Community interactions have a significant positive impact on commercial ice rink consumers’ repurchase intention*.

**Hypothesis** **10.** *Community interactions have mediation effect between perceived functional, social, emotional, and risk values and commercial ice rink consumers’ repurchase intention*.

**Figure 1:**
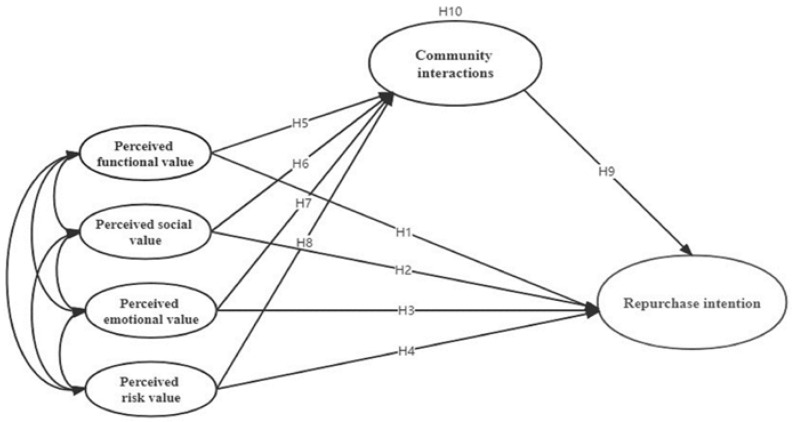
Structural equation model.

In summary, we constructed a mediation effect model between perceived value, community interactions, and commercial ice rink consumers’ repurchase intention (Figure 1).

## 2. Research Methods

### 2.1. Data Collection

To verify the hypotheses, we adopted questionnaire surveys and collected data through online and offline methods, which is reasonable in some references [50,51]. Electronic questionnaires were distributed through WeChat groups of commercial ice rink consumers (one-month duration), while printed questionnaires were distributed through field visits to 11 commercial ice rinks in six cities. A total of 400 questionnaires were returned, of which 347 were valid, with an effective rate of 86.8% (in the questionnaire survey, consumers who repeatedly consume 2 times or more were selected as basic questionnaires. Meanwhile, some questionnaires with fast response time and regular distribution of answers were eliminated) (Table 1). According to Zhang and Dong [52], the sample size should be more than 20 times the number of independent variables. Therefore, this sample size fits the requirement of the research.

### 2.2. Variables Measurement

Perceived value, community interactions, and consumers’ repurchase intention are latent variables. We designed a questionnaire based on sorting through existing literature scales (Table 2). In view of the contents’ integrality of latent variables and ease for Chinese consumers to understand, we revised the expression of individual statements.

In measuring perceived value, we adopted a revised questionnaire by Du and Deng for perceived functional value [45], which mainly measures consumer perception of commercial ice rink service quality and improvement in personal ability. A revised questionnaire by Perez et al. was used for measuring perceived social value [53], which measures consumer perception of the social responsibility of commercial ice rinks. A questionnaire by Kim et al. was adopted [54] for measuring the perceived emotional value that focuses on measuring emotional experience, psychological state, and satisfaction with commercial ice rinks. Perceived risk value was measured based on Bauer’s [55] questionnaire, covering various risks related to physical activity, privacy, bundled consumption, and pricing.

We adopted the scale by Yang [56] to measure community interactions via interactivity and enthusiasm among consumer communities.

Consumers’ repurchase intention was measured using an adapted scale of Chiu et al. [57]. Furthermore, we assigned consumer innovation as a label variable to mitigate the impact of common method biases influencing the research results [58]. All the questionnaires were based on a 7-point Likert scale ranging from “strongly disagree” (1) to “strongly agree” (7).

### 2.3. Data Processing

We initially conducted a reliability and validity analysis to crosscheck the collected data. The Cronbach’s alpha coefficients of all the latent variables were greater than 0.847, indicating good reliability. Confirmatory factor analysis (CFA) showed that the average variance extraction (AVE) and composite reliability (CR) of each latent variable met critical conditions, indicating that the latent variables had excellent convergent validity (Table 3). Moreover, the square roots (diagonal values) of the AVE of all latent variables were greater than the correlation coefficient between latent variables, indicating solid discriminant validity between latent variables (Table 4).

Concurrently, we adopted the label variable method of Tang and Wen to verify common method biases [59]. No significant difference between the baseline model (no correlation between the label and latent variables) and the U model (introduction of label variables) was found (Table 5). The verification proved the absence of significant common method biases.

## 3. Empirical Results

### 3.1. Descriptive Statistics

Table 6 shows that the descriptive statistical results showed specific correlations among perceived functional, social, emotional, and risk values, community interactions, and the repurchase intention of commercial ice rink consumers, laying the foundation for analyzing the research hypotheses.

### 3.2. Structural Equation Analysis

**Figure 2:**
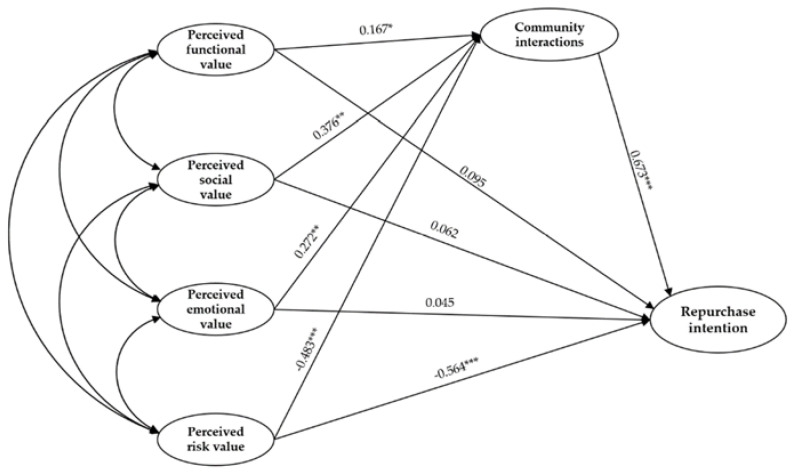
The results of the SEM analysis. *** *p* < 0.001, ** *p* < 0.01, * *p* < 0.05.

Table 7 shows that the structural equation model fit indices (χ^2^/df = 2.345, RMSEA = 0.062, CFI = 0.963, IFI = 0.963, TLI = 0.957, NFI = 0.938, SRMR = 0.036, GFI = 0.887) were consistent with the relevant indicators required [60]. The results show that perceived risk value significantly negatively impacts community interactions and repurchase intention; perceived functional, social, and emotional values significantly impact community interactions positively without significantly impacting repurchase intention. Community interactions have a significant positive impact on repurchase intention. Consequently, H1–H3 are not supported, and H4–H9 are supported (Figure 2).

### 3.3. Mediation Effect Test

We applied Amos 27.0 software and bootstrapping technology [61] to further analyze the mediation effect of community interactions on perceived functional, emotional, social perception, and risk values and commercial ice rink consumers’ repurchase intention (Table 8). The results show a significant mediation effect of community interactions between perceived risk value and consumers’ repurchase intention (95% of the confidence interval is −1.177~−0.613), and the value of the mediation effect is −0.889. Community interactions are partially mediating since perceived risk value directly impacts consumers’ repurchase intention. It is evident that the negative impact of perceived risk value on community interactions further hinders the repurchase intention.

Community interactions mediate between consumers’ perceived emotional value and repurchase intention (95% of the confidence interval is within 0.042–0.395), including consumers’ perceived social value and repurchase intention (95% of the confidence interval is within 0.046–0.596). The values of mediation effects are 0.183 and 0.253. Simultaneously, since perceived emotional and social values have no significant direct impact on consumers’ repurchase intention, community interactions fully mediate between perceived emotional and social values and consumers’ repurchase intention, showing that perceived emotional and social values ultimately impact repurchase intention through community interactions.

Additionally, community interactions do not mediate between consumers’ perceived functional value and repurchase intention (95% the confidence interval is −0.035–0.315).

## 4. Discussion

### 4.1. Results and Discussion

Firstly, perceived functional value has a significant positive impact on community interactions but has no significant effect on repurchase intention. This is not entirely consistent with existing research conclusions [21]. Although having a more comprehensive perception of the functions and services of ice rinks impels consumers to interact, it does not necessarily translate into repurchase intention. Commercial ice rinks’ over-reliance on a single sports activity such as ice skating no longer satisfies customers. Many people have shifted their attention to other ice-based sports. Additionally, the diverse factors commercial ice rinks employ to attract consumers are far beyond the limitations of perceived functional value, neutralizing its impact on consumers’ repurchase intention.

Secondly, perceived social value has a significant positive impact on community interactions but has no significant positive effect on repurchase intention, and community interactions fully mediate perceived social value and repurchase intention. Consumers will form a more favorable social perception when they perceive the solid social responsibility of an ice rink, increasing their inclination to receive social behavior information of enterprises in the community and interact, which catalyze their repurchase intention.

Thirdly, perceived emotional value significantly and positively affects community interactions but does not substantially impact repurchase intention. Community interactions fully mediate customers’ perceived emotional value and repurchase intention. A commercial ice rink community’s stability and interactivity are essential for converting perceived emotional value into repurchase intention. Community interactions act like perceived emotional value glue that binds the perception information of different consumers in the interaction process, enabling consumers to better understand the products and their service preferences and make choices. Such enhanced consumer preference significantly boosts member loyalty, translating into repurchase intention. Essentially, the perceived emotional value does not directly increase customer repurchase intention; this can only be through community interactions.

Finally, perceived risk value has a significant negative impact on repurchase intention. It indirectly influences repurchase intention through the mediation of community interactions, creating a partial mediation effect consistent with existing research [32,43]. Commercial ice rink operators are faced with the immediate challenge of establishing a more reliable risk prevention and control mechanism to ensure customer safety.

### 4.2. Practical Implication

Based on the research conclusions, we propose the following countermeasures to increase consumer repurchase intention.

1. Extending commercial ice rink product lines. Based on the role of perceived value through the above empirical analysis, building diversified product lines is very important. Firstly, more diverse sports activities should be provided through improving the utilization rate of the ice rink. Secondly, strenuous efforts can be made in event planning, media promotions, business development, etc., hosting community, campus, and other events to cater to different groups, and cooperating with equipment manufacturing companies, sponsors at all levels, and sports brokerage companies to comprehensively enhance the influence of the events and meet consumers’ needs. Thirdly, product line extension can also include the active practice of integrating sports and education, creating ice summer school camp services, reinforcing consumers’ perception of the function of commercial ice rinks, and intensifying the student community’s enthusiasm for ice sports and participation through a centralized training model.

2. Creating a commercial ice rink intellectual property (IP) system. The empirical analysis confirms the importance of community interactions, which is inseparable from consumption experience. In order to form favorable consumption experience, it is crucial to build unique intellectual property (IP) of the commercial ice rink. First, a commercial ice rink IP space can transform the ice rink into a platform for disseminating excellent culture, promoting cultural exchanges, and creating consumption and sports experience that integrates culture, art, technology, lifestyles, and aesthetics for consumers, encouraging social group expansion. Second, accelerating the research and development of IP peripheral products and developing IP-related derivatives around commercial ice rink IP culture, including ice sportswear, equipment, related gifts, souvenirs, and accessories to amplify coverage and impact. Third, ice rink operators may actively organize various events at all levels, such as figure skating and curling and selling naming rights to well-known brands.

3. Improving consumer sports career planning. Ice rink operators are advised to adhere to a “two-wheel-drive” tenet of popularity and professionalism based on the analysis results of perceived emotional value. To enhance consumers’ emotional perception, operators need to establish sports career plans for different groups of patrons, create high-quality, diversified services and marketing, and ensure and maintain positive community interactions to increase repurchase intentions. Adopting the people-oriented planning concept is crucial for business success, calling for attention to both ability and psychological building in consumers’ sports career planning, and facilitating communication between consumers and coaches. The focus needs to be on enhancing consumers’ sense of gain while considering all-around business development.

4. Enhancing risk management. Risk prevention and control and emergency plans should be systemized in view of the importance of perceived risk value. Ice rinks should regularly conduct self-inspections of infrastructures and emergency drills, raise consumers’ safety awareness through lectures, training videos, etc., and fulfill safety obligations by making safety facilities, officers, and volunteers available. Further, scientific training methods should be adopted. Ice sports have strict requirements for bones and strength, and issues such as whether a consumer is fit for going on the ice and the choice of training methods should be addressed. Technologies can be introduced for physical condition monitoring and assisting consumers in choosing appropriate training methods combined with comprehensive assessments by coaches to minimize risks and increase repurchase intentions.

## 5. Conclusions

We analyzed the mechanisms that affect the repurchase intention of commercial ice rink consumers and the mediating role of community interactions between perceived value and repurchase intention based on the theory of perceived value and drew the following conclusions. The results show that consumers’ perceived risk value significantly impacts community interactions and consumers’ repurchase intention. Perceived functional, social, and emotional values positively affect community interactions but have an insignificant impact on the consumers’ repurchase intention. Community interactions play a full mediation role between perceived emotional and social values and consumers’ repurchase intention; they play partially mediating role between consumers’ perceived risk value and repurchase intention and do not have mediating role between perceived functional value and consumers’ repurchase intention. These conclusions provide some practical reference for promoting the development of commercial ice stadia; for instance, extending commercial ice rink product lines, creating a commercial ice rink intellectual property (IP) system, improving consumer sports career planning, and enhancing risk management.

This study may have a few limitations. Firstly, most of the data collected came from consumers in China’s first-tier cities, which means that the results are more applicable to commercial ice rink consumers in China’s economically developed regions. This can be improved in the future by collecting samples from the “sinking market” (Chinese concept of third-tier cities and below) and conducting a comparative analysis. Secondly, the repurchase intention of commercial ice rink consumers is based on the theoretical framework of developed Western countries. Research based on theories more relevant to Chinese consumers’ repurchase intention is needed in the future, as consumer behavior tends to be influenced by local culture and economic conditions. Therefore, exploring the impact of other variables on consumers’ repurchase intention, e.g., the culture and operation model of a commercial ice rink, will be very important through multilevel analysis.

## Figures and Tables

**Table 1:** Demographic information of the survey sample.

Category	Percentage (%)	Category	Percentage (%)
Gender	−	Income level	−
Male	56.06%	Below 2000	3.03%
Female	43.94%	2001~4000	6.31%
Age	−	4001~6000	16.42%
Under 18 years old	1.77%	6000~8000	16.92%
18~25	22.22%	8000~10,000	16.16%
26~30	30.30%	10,000~20,000	24.24%
31~40	39.14%	20,000~50,000	15.91%
41~50	5.56%	50,000 or more	1.01%
Above 50	1.01%	Commercial ice rink consumption frequency	−
Education level	−	Less than once a week	20.20%
High school and below	9.34%	1–3 times a week	43.94%
Junior college	13.89%	4–6 times per week	35.10%
Bachelor	43.69%	7 times a week and above	0.76%
Master and above	33.08%	−	−

**Table 2:** The items of latent variables.

Variables	Items	References
Perceived functional value	1. I can improve my sports skills at the ice rink	Du and Deng [45]
	2. I think I can make the best use of my fragmented time at the ice rink	
	3. The service of the ice rink is professional and comprehensive	
	4. I am satisfied with the overall service quality of the ice rink	
Perceived social value	1. My peers around me be willing to spend money at the ice rink	Perez et al.[53]
	2. I think I should go to the ice rink because everyone seems to go there	
	3. People around me who are influential or important to me recommend that I go to the ice rink	
	4. It has become a trend to go to the ice rink	
Perceived emotional value	1. I don’t think my time at the rink is wasted	Kim et al. [54]
	2. I feel relaxed when I exercise in the ice rink	
	3. The ice rink adds joy to my life	
	4. I enjoy the process of sport at the ice rink	
Perceived risk value	1. I am worried about the safety risks of sport at the ice rink	Bauer [55]
	2. I am worried that my privacy will be compromised by the ice rink operator	
	3. I am worried that there will be bundled purchases after spending money at the ice rink	
	4. I am worried that the pricing of the ice rink operator’s program is not transparent, resulting in unclear economic losses	
Community interactions	1. I often discuss information and knowledge about ice sports with other customers at the ice rink	Yang [56]
	2. I have common opinions with some customers of the ice rink and we can talk together	
	3. I can cooperate with other customers at the ice rink to diagnose and solve product/sport problems	
	4. Communication with other customers at the ice rink is pleasant	
	5. The usage behavior of others does not influence my willingness to spend money at the ice rink	
Repurchase intention	1. I would like to continue to consume at the ice rink if I can	Chiu [57]
	2. I have the intention to maintain continuous consumption at the ice rink	
	3. I may participate in more projects at the ice rink in the future	
	4. I will not spend money at the ice rink in the future	
Consumer innovation (label variable)	1. I like to pursue new technologies and things	Cha [58]
	2. I usually buy new products earlier than others	
	3. I believe that the progress of technology will result in a better life	
	4. I am willing to accept all kinds of novelties in my life	

**Table 3:** Analysis of reliability and convergent validity.

Variables	α	CR	AVE
Perceived functional value	0.847	0.848	0.583
Perceived social value	0.899	0.904	0.702
Perceived emotional value	0.893	0.893	0.676
Perceived risk value	0.907	0.910	0.717
Community interactions	0.956	0.957	0.847
Repurchase intention	0.974	0.975	0.928
Label variable	0.893	0.893	0.677

**Table 4:** Analysis of discriminant validity.

Construct Face	Perceived Functional Value	Perceived Social Value	Perceived Emotional Value	Perceived Risk Value	Community Interactions	Repurchase Intention	Labeling Variables
Perceived functional value	0.763	−	−	−	−	−	−
Perceived social value	0.706	0.838	−	−	−	−	−
Perceived emotional value	0.751	0.780	0.822	−	−	−	−
Perceived risk value	−0.57	−0.714	−0.644	0.847	−	−	−
Community interactions	0.671	0.764	0.739	−0.765	0.920	−	−
Repurchase intention	0.613	0.7	0.666	−0.779	0.824	0.963	−
Label variable	−0.112	−0.104	−0.099	−0.131	−0.073	0.044	0.823

**Table 5:** Fitting results and comparison of each model.

Number	Model	χ^2^/df	RMSEA	CFI	IFI	TLI	NFI	SRMR	GFI	Compare	∆χ^2^	∆df
CFA	CFA model	1.946	0.052	0.967	0.967	0.962	0.934	0.338	0.888	−	−	−
B	Baseline B model	1.914	0.051	0.967	0.967	0.963	0.934	0.048	0.887	B vs U	18.888	23.000
U	U model	2.001	0.054	0.966	0.967	0.959	0.936	0.033	0.890	−	−	−

**Table 6:** The results of descriptive statistics.

Variable Name	Mean	Standard Deviation	Perceived Functional Value	Perceived Social Value	Perceived Emotional Value	Perceived Risk Value	Community Interactions	Repurchase Intention
Perceived functional value	6.180	0.868	−	−	−	−	−	−
Perceived social value	4.825	0.806	0.605 **	−	−	−	−	−
Perceived emotional value	4.456	0.863	0.651 **	0.704 **	−	−	−	−
Perceived risk value	1.873	0.874	−0.510 **	−0.662 **	−0.590 **	−	−	−
Community interactions	6.203	1.092	0.605 **	0.712 **	0.684 **	−0.731 **	−	−
Repurchase intention	5.733	1.518	0.558 **	0.663 **	0.618 **	−0.747 **	0.795 **	−

Note: ** *p* < 0.01.

**Table 7:** The results of empirical analysis.

	Hypothesis	Unstandardized Coefficients	S.E.	C.R.	*p*	Results
H1	Perceived functional value → repurchase intention	0.095	0.108	0.879	0.379	Not supported
H2	Perceived social value → repurchase intention	0.062	0.154	0.399	0.690	Not supported
H3	Perceived emotional value → repurchase intention	0.045	0.120	0.375	0.708	Not supported
H4	Perceived risk value → repurchase intention	−0.564	0.095	−5.94	***	Supported
H5	Perceived functional value → Community interactions	0.167	0.084	1.986	0.047 *	Supported
H6	Perceived social value → community interactions	0.376	0.119	3.15	0.002 **	Supported
H7	Perceived emotional value → Community interactions	0.272	0.093	2.936	0.003 **	Supported
H8	Perceived risk value → Community interactions	−0.483	0.066	−7.314	***	Supported
H9	Community interactions → repurchase intention	0.673	0.088	7.679	***	Supported

Note: Goodness-of-fit indicators χ^2^/df < 3, GFI > 0.9, comparative fitness indicators (NFI, IFI, TLI, CFI) > 0.9, SRMR < 0.05, and RMSEA < 0.08; *** *p* < 0.001, ** *p* < 0.01, * *p* < 0.05.

**Table 8:** The analytical results of mediating effects.

Path		Point Estimate	Coefficient Product		Bootstrapping			
					Bias-Corrected Percentile 95% CI		Percentile 95% CI	
			SE	Z	Lower	Upper	Lower	Upper
Total effect	PRV → RI	−0.889	0.145	−6.131	−1.177	−0.613	−1.171	−0.609
	PEV → RI	0.228	0.165	1.382	−0.076	0.553	−0.096	0.537
	PSV → RI	0.315	0.235	1.340	−0.141	0.783	−0.114	0.835
	PFV → RI	0.207	0.180	1.150	−0.159	0.550	−0.148	0.553
Direct effects	PRV → RI	−0.564	0.158	−3.570	−0.946	−0.306	−0.915	−0.298
	PEV → RI	0.045	0.123	0.366	−	−	−	−
	PSV → RI	0.062	0.225	0.276	−	−	−	−
	PFV → RI	0.095	0.194	0.490	−	−	−	−
Indirect effects	PRV → RI	−0.325	0.078	−4.167	−0.497	−0.188	−0.472	−0.163
	PEV → RI	0.183	0.090	2.033	0.042	0.395	0.035	0.382
	PSV → RI	0.253	0.142	1.782	0.046	0.596	0.040	0.574
	PFV → RI	0.112	0.084	1.333	−0.035	0.315	−0.064	0.278

Note: PRV = perceived risk value; PEV = perceived emotional value; PSV = perceived social value; PFV = perceived functional value; RI = repurchase intention.

## Data Availability

The data that support the findings of this study are available on request from the corresponding author.
